# Assessment of TyG Index and Modified TyG Indices in Type 2 Diabetes Mellitus: Evaluating Their Potential as Predictors of Glycemic Control

**DOI:** 10.7759/cureus.80785

**Published:** 2025-03-18

**Authors:** Vedika Rathore, Kapila Gaikwad, Roshan K Mahat

**Affiliations:** 1 Department of Biochemistry, Shyam Shah Medical College, Rewa, IND; 2 Department of Biochemistry, Dharanidhar Medical College and Hospital, Keonjhar, IND

**Keywords:** cross-sectional study, glycemic control, modified tyg indices, triglyceride glucose, tyg index, type 2 diabetes mellitus

## Abstract

Introduction

The triglyceride glucose (TyG) index and modified TyG-indices have been suggested as a reliable indication of insulin resistance. The present study aimed to investigate the predictive utility of TyG index and modified TyG indices (TyG-waist circumference, TyG-body mass index, TyG-waist-to-hip ratio, and TyG-waist-to-height ratio) for assessing glycemic control in type 2 diabetes mellitus (T2DM).

Methods

The present hospital-based cross-sectional study recruited 383 T2DM patients. On the basis of HbA1c levels, patients were grouped into poor glycemic control (n=168) and good glycemic control (n=215). Baseline and biochemical parameters including TyG and modified TyG indices were compared between the groups. We used a Spearman correlation analysis to look for an association between TyG and TyG-related indices and glycemic control. We conducted receiver operating characteristic curve analysis to evaluate the predictive capability of TyG-index and modified TyG indices in assessing poor glycemic control in T2DM.

Results

T2DM with poor glycemic control had significantly elevated TyG and modified TyG indices when compared to those with good glycemic control. The TyG index and modified TyG indices showed a strong correlation with glycemic control in individuals with T2DM. The TyG index exhibited greater predictive capacity for poor glycemic control as compared to the modified TyG indices.

Conclusion

Patients with T2DM who are treated in clinical settings with limited resources may benefit from using the TyG index to evaluate their glycemic control.

## Introduction

Type 2 diabetes mellitus (T2DM) is a prevalent metabolic disorder characterized by abnormalities in blood glucose metabolism, leading to chronic hyperglycemia and an increased risk of severe complications [1]. The global incidence of diabetes has risen significantly due to factors such as aging populations, rising obesity rates, and lifestyle changes. In 2021, approximately 500 million adults aged 20 to 79 were diagnosed with diabetes mellitus, with projections estimating that this number will exceed 600 million by 2030 and reach 783 million by 2045. Furthermore, diabetes and its complications accounted for an estimated 6.7 million deaths worldwide in 2021 [2]. Poor glycemic control accelerates the progression of diabetes, increasing the risk of both macrovascular and microvascular complications that can significantly reduce life expectancy, diminish quality of life, and elevate healthcare costs [3]. However, maintaining optimal glycemic control has been shown to improve patients’ morbidity, life expectancy, and overall standard of living [4].

The primary therapeutic goal in T2DM management is to achieve and sustain glycemic control to prevent diabetes-related complications [5]. Glycemic control in individuals with T2DM is commonly assessed using three key biomarkers: glycated hemoglobin (HbA1c), fasting plasma glucose (FPG), and postprandial glucose (PPG). Among these, HbA1c is widely regarded as the most reliable measure, reflecting average blood glucose levels over approximately three months [6]. While HbA1c testing is a cornerstone in diabetes management, its frequency is determined by clinical context, treatment strategy, and healthcare provider discretion [7]. However, in resource-limited settings, frequent laboratory testing is often constrained by high costs and a lack of standardized assays.

Insulin resistance (IR) plays a crucial role in the pathophysiology of diabetes, directly influencing glycemic control. Researchers have focused on improving insulin sensitivity to mitigate diabetes-related complications and associated chronic conditions [8]. The hyperinsulinemic-euglycemic clamp (HIEC) is considered the gold standard for assessing IR [9]; however, it requires specialized infusion equipment, frequent blood glucose monitoring, and advanced technological resources, making it impractical for routine clinical use [10]. The homeostasis model assessment of insulin resistance (HOMA-IR) is commonly used in clinical settings, as it estimates IR based on fasting insulin and glucose levels [11]. However, the requirement for plasma insulin or C-peptide assays makes HOMA-IR costly, less accessible in many laboratories, and limited by low reproducibility.

Given these limitations, the triglyceride glucose (TyG) index has emerged as a simpler and cost-effective alternative for evaluating IR. The TyG index is derived from FPG and triglyceride levels and has demonstrated superior predictive ability for IR compared to HOMA-IR [12] and HIEC [13]. Additionally, modified TyG indices incorporating anthropometric parameters, such as TyG-waist circumference (TyG-WC), TyG-body mass index (TyG-BMI), and TyG-waist-to-height ratio (TyG-WHtR), have been proposed as surrogate markers for IR and metabolic dysfunction [14].

While the TyG index and its modified variants have been associated with IR, limited studies have explored their diagnostic efficacy in assessing glycemic control in T2DM patients [15-17]. Maintaining optimal glycemic control is crucial in preventing diabetes-related complications. In light of the necessity for accessible and cost-effective diagnostic tools, it is imperative to investigate the predictive performance of the TyG index and its modified variants in the assessment of glycemic control. We hypothesize that a higher TyG index and its modified variants will correlate with poorer glycemic control, as indicated by elevated HbA1c levels. Moreover, elucidating the comparative effectiveness of these indices in predicting glycemic control could significantly enhance clinical decision-making, particularly within resource-limited settings. Thus, the present study aims to evaluate the predictive performance of the TyG index and modified TyG indices (TyG-WC, TyG-BMI, TyG-waist-to-hip ratio [TyG-WHR], and TyG-WHtR) in determining glycemic control among individuals with T2DM.

## Materials and methods

Study participants

This hospital-based cross-sectional study was undertaken in the Department of Biochemistry, Shyam Shah Medical College, Rewa, Madhya Pradesh, India, over a period of 10 months (from December 2022 through September 2023). This study recruited 383 T2DM patients from the outpatient department of Medicine at Shyam Shah Medical College, Rewa. T2DM was established according to the standards outlined by the American Diabetes Association (ADA) [18]. T2DM patients had FPG ≥126 mg/dL and/or HbA1c ≥6.5%. The necessary sample size for the current study was determined using the standard formula, taking into account the prevalence for a single population. The formula used was n = z2p(1-p)/e2, assuming standard normal variables (z score) of 1.96 at a 95% confidence interval, a margin of error (e) of 5%, and a prevalence (p) of 46.43% for poor glycemic control, as reported in the study conducted by Selvi et al. in India in 2021 [16]. Study participants were categorized into two groups based on their HbA1c values. Therefore, HbA1c levels below 7.0% were categorized as good glycemic control (n=215), while levels ≥7.0% were categorized as poor glycemic control (n=168).

Participants were excluded if they had type 1 diabetes, cancer, Cushing's syndrome, thyroid disorders, hypertension; were receiving systemic corticosteroid treatment, using lipid-lowering medications; were diagnosed with viral hepatitis (acute or chronic), liver, renal, or heart failure; were experiencing infection or inflammation; or were pregnant. Demographic information, anthropometric measurements, clinical information, family history of diabetes, and duration of diabetes were documented.

The protocol of the present study received approval from the Institutional Ethics Committee of Shyam Shah Medical College, Rewa (Reference No: IEC/MC/2021/26837, dated: 30-11-2021). Each participant signed an informed consent form for the study. The Helsinki Declaration was followed, ensuring all human rights were protected.

Anthropometric data and biochemical measurements

Anthropometric measurements were taken with individuals wearing light clothing. Height was measured to the nearest 0.1 cm and body weight to the nearest 0.1 kg. Body mass index (BMI) was computed as weight (in kg) divided by height (in m^2^). A measuring tape without stretchability was employed to measure waist circumference (WC) and hip circumference (HC). The measurement of WC was done around the midpoint between the lower margin of the last palpable rib and the top of the iliac crest. The measurement of HC was taken at levels of the greater trochanter. The waist-to-hip ratio (WHR) was determined by dividing the WC (cm) by the HC (cm). Systolic blood pressure (SBP) and diastolic blood pressure (DBP) were then determined using a conventional sphygmomanometer following established protocols. A total of 5 mL of blood was obtained from each patient after they had fasted overnight. Levels of FPG and lipid profiles such as total cholesterol (TC), triglycerides (TG), and high-density lipoprotein-cholesterol (HDL-C) were assessed using commercially accessible kits on an automated clinical chemistry analyzer (Model XL-1000, Transasia Bio-Medicals Ltd., New Delhi, India). HbA1c levels were measured in whole blood using immunoturbidimetric method on an automated clinical chemistry analyzer (Model XL-1000, Transasia Bio-Medicals Ltd). Low-density lipoprotein cholesterol (LDL-C) and very low-density lipoprotein cholesterol (VLDL-C) were determined using the Friedewald equation.

The TyG index and modified TyG indices were computed by utilizing the following formula:

1) TyG index =Ln [fasting triglycerides (mg/dL) × fasting glucose (mg/dL)]/2 [19].

2) TyG-BMI =TyG index × BMI

3) TyG-WC =TyG index × WC

4) TyG-WHR =TyG index × WHR

5) TyG-WHtR = TyG index × WHtR

Statistical analysis

Statistical Package for Social Science Version 20 for Windows (IBM Corp., Armonk, NY, USA) and MedCalc statistical software Version 22.021 were used to analyze the data. The data's normality was evaluated using the Kolmogorov-Smirnov test. Variables were presented as mean ± standard deviation (SD) for those following a normal distribution and as median (Interquartile range, IQR) for those not following a normal distribution. The two groups (poor glycemic control and good glycemic control) were compared using the Student t-test for normally distributed variables and the Mann-Whitney U-test for non-normally distributed variables. The chi-square test was employed to evaluate categorical variables. Receiver operating characteristic (ROC) curve analysis was conducted to assess the predictive capability of TyG-index and modified TyG indices for poor glycemic control. The optimal cut-off value was identified based on the point with the maximum Youden index. A p-value of <0.05 was considered statistically significant.

## Results

Table 1 shows the baseline and biochemical characteristics of the studied subjects. Of 383 T2DM, 168 (43.86%) patients had a poor glycemic control. The patients’ mean age was 46.98 years. The age, gender, duration of diabetes, SBP, DBP, BMI, TG, and VLDL-C of T2DM patients with poor glycemic control and T2DM patients with good glycemic control were not statistically significantly different. T2DM patients with poor glycemic control exhibited significantly higher values for WC, WHR, FPG, HbA1c, TC, LDL-C, TyG index, TyG-BMI, TyG-WC, TyG-WHR, and TyG-WHtR compared to T2DM patients with good glycemic control. However, in comparison with good glycemic control patients, poor glycemic control patients had significantly low levels of HDL-C.

**Table 1:** Baseline and biochemical characteristics of studied subjects. *Significant at p<0.05. Data are presented as mean ± SD for normally distributed variables and as median (IQR) for non-normally distributed variables. The Student independent t-test was used for normally distributed continuous variables, the Mann-Whitney U test (U) was used for non-normally distributed continuous variables, and the chi-square test (χ²) was used for categorical variables. T2DM, type 2 diabetes mellitus; SBP, systolic blood pressure; DBP, diastolic blood pressure; BMI, body mass index; WC, waist circumference; WHR, waist-to-hip ratio; FPG, fasting plasma glucose; HbA1c, glycated hemoglobin; TC, total cholesterol; TG, triglyceride; HDL-C, high-density lipoprotein cholesterol; LDL-C, low-density lipoprotein cholesterol; VLDL-C, very low-density lipoprotein cholesterol; TyG index, triglyceride glucose index; TyG-BMI, product of TyG index and body mass index; TyG-WC, product of TyG index and waist circumference; TyG-WHR, product of TyG index and waist-to-hip ratio; TyG-WHtR, product of TyG index and waist-to-height ratio

Variables	Total T2DM (n=383)	Poor glycemic control (n=168)	Good glycemic control (n=215)	Test statistics value	p-Value
Age (years)	46.98 ± 6.54	47.14±7.57	46.85±5.63	t = 0.42	0.677
Sex (male/female)	217/166	97/71	120/95	χ² = 0.1422	0.706
Duration of T2DM (months)	38.11 ± 17.6	39.92 ± 19.43	36.7 ± 15.92	t = 1.74	0.083
SBP (mmHg)	122.94 ± 6.79	123.61 ± 7.6	122.42 ± 6.04	t = 1.65	0.10
DBP (mmHg)	82 (7)	83 (6)	82 (7)	U = 16895	0.279
BMI (kg/m^2^)	27.16 (3.69)	27.17 (4.01)	26.87 (3.69)	U = 17676	0.721
WC (cm)	90 (15)	91 (16)	90 (16)	U = 15704.5	0 .029^*^
WHR	0.92 (0.06)	0.93 (0.06)	0.92 (0.06)	U = 10411	<0.001^*^
FPG (mg/dL)	168 (39.5)	196.4 ± 19.72	153.98 ± 14.28	t = 23.48	<0.001^*^
HbA1c (%)	6.88 (3.16)	9.94 ± 1.35	6.5 ± 0.31	t = 32.49	<0.001^*^
TC (mg/dL)	197.11 ± 16.85	200.92 ± 14.3	194.13 ± 18.09	t = 4.1	<0.001^*^
TG (mg/dL)	177.46 ± 16.42	179.07 ± 18.47	176.2 ± 14.55	t = 1.65	0.10
HDL-C (mg/dL)	41.54 ± 5.81	39.78 ± 5.5	42.92 ± 5.68	t = 5.47	<0.001^*^
LDL-C (mg/dl)	120.08 ± 18.92	125.32 ± 16.12	115.97 ± 19.95	t = 5.07	<0.001^*^
VLDL-C (mg/dL)	35.49 ± 3.28	35.81 ± 3.69	35.24 ± 2.91	t = 1.65	0.10
TyG index	5.16 ± 0.09	5.23 ± 0.08	5.1 ± 0.06	t = 17.23	<0.001^*^
TyG-BMI	138.47 ± 16.19	140.9 ± 17.02	136.58 ± 15.29	t = 2.61	0.009^*^
TyG-WC	462.06 (81.35)	469.39 (86.01)	460.13 (84.34)	U = 13319	<0.001^*^
TyG-WHR	4.68 ± 0.25	4.81 (0.33)	4.64 (0.34)	U = 8233	<0.001^*^
TyG-WHtR	2.67 (0.54)	2.77 (0.58)	2.62 (0.48)	U = 13642	<0.001^*^

The TyG index and TyG-related indices showed a positive and significant correlation with glycemic control (HbA1c) in all T2DM (p<0.001 for all). Among all indices, the TyG index showed a very strong correlation with glycemic control, as shown in Table 2.

**Table 2:** Correlation of TyG and modified TyG indices with glycemic control in all patients. *Significant at p<0.05; ρ, Spearman's rank correlation coefficient. TyG index, triglyceride glucose index; TyG-BMI, product of TyG index and body mass index; TyG-WC, product of TyG index and waist circumference; TyG-WHR, product of TyG index and waist-to-hip ratio; TyG-WHtR, product of TyG index and waist-to-height ratio

Variables	ρ	p-Value
TyG index	0.77	<0.001^*^
TyG-BMI	0.26	<0.001^*^
TyG-WC	0.29	<0.001^*^
TyG-WHR	0.54	<0.001^*^
TyG-WHtR	0.27	<0.001^*^

**Figure 1:**
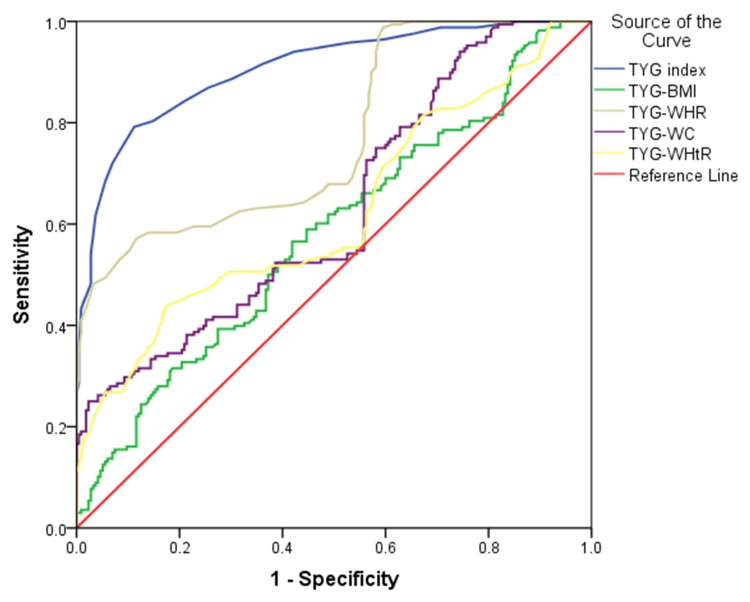
ROC curve analysis for TyG index and modified TyG indices in predicting poor glycemic control in T2DM TyG index, triglyceride glucose index; TyG-BMI, product of TyG index and body mass index; TyG-WC, product of TyG index and waist circumference; TyG-WHR, product of TyG index and waist-to-hip ratio; TyG-WHtR, product of TyG index and waist-to-height ratio; ROC, receiver operating characteristic; TyG, triglyceride glucose; T2DM, type 2 diabetes mellitus

Figure 1 shows the results of the ROC curve analysis for the TyG index and modified TyG indices in predicting poor glycemic control in T2DM.

Table 3 presents the optimal cut-off values, Youden index, sensitivity, specificity, and area under the curve (AUC) with 95% confidence intervals for the TyG index and its modified indices in identifying poor glycemic control in individuals with T2DM. The TyG index demonstrated the highest discriminatory ability, with an optimal cut-off value of 5.18, yielding a sensitivity of 79.2% and specificity of 88.8% (AUC = 0.906, 95% CI: 0.876-0.936, p = 0.000). Among the modified indices, the TyG-WHR exhibited the highest AUC (0.772, 95% CI: 0.725-0.820, p = 0.000), with a cut-off value of 4.78, sensitivity of 57.1%, and specificity of 88.4%. The TyG-WC index had a relatively low sensitivity (25%) but a high specificity (97.7%), with an AUC of 0.631 (95% CI: 0.575-0.687, p = 0.000). Similarly, the TyG-WHtR index had an AUC of 0.622 (95% CI: 0.565-0.680, p = 0.000), with a cut-off value of 2.83, sensitivity of 44%, and specificity of 82.3%. The TyG-BMI index exhibited the lowest AUC (0.579, 95% CI: 0.522-0.637, p = 0.008), with a cut-off value of 140.32, sensitivity of 56.5%, and specificity of 58.1%. These findings suggest that the TyG index has the strongest predictive ability for poor glycemic control in T2DM, while the modified indices show varying degrees of diagnostic performance, with TyG-WHR demonstrating relatively better accuracy among them.

**Table 3:** Optimal cut-off values, sensitivity, specificity, and AUC of the TyG index and modified TyG indices *Significant at p<0.05 TyG index, triglyceride glucose index; TyG-BMI, product of TyG index and body mass index; TyG-WC, product of TyG index and waist circumference; TyG-WHR, product of TyG index and waist-to-hip ratio; TyG-WHtR, product of TyG index and waist-to-height ratio; AUC, area under the curve; CI, confidence interval

Variables	Youden index (J)	cut-off value	Sensitivity (%)	Specificity (%)	AUC	95% CI		p-value
						Lower bound	Upper bound	
TyG index	0.68	5.18	79.2	88.8	0.906	0.876	0.936	0.000^*^
TyG-BMI	0.15	140.32	56.5	58.1	0.579	0.522	0.637	0.008^*^
TyG-WC	0.23	506.24	25	97.7	0.631	0.575	0.687	0.000^*^
TyG-WHR	0.46	4.78	57.1	88.4	0.772	0.725	0.820	0.000^*^
TyG-WHtR	0.26	2.83	44	82.3	0.622	0.565	0.680	0.000^*^

We also conducted a comparison of the differences in the AUC of the TyG index and modified TyG indices. Table 4 clearly indicates that the AUC for TyG index was significantly distinct from the AUC of other TyG-related indices.

**Table 4:** Pairwise comparison of AUC of TyG index with modified TyG indices *Significant at p<0.05 TyG index, triglyceride glucose index; TyG-BMI, product of TyG index and body mass index; TyG-WC, product of TyG index and waist circumference; TyG-WHR, product of TyG index and waist-to-hip ratio; TyG-WHtR, product of TyG index and waist-to-height ratio; AUC, area under the curve; CI, confidence interval

Variables	Differences between AUC	95% CI	z statistic	P-Value
TyG-BMI	0.327	0.268 to 0.385	10.968	< 0.001^*^
TyG-WC	0.275	0.214 to 0.335	8.919	< 0.001^*^
TyG-WHR	0.134	0.0847 to 0.183	5.324	< 0.001^*^
TyG-WHtR	0.284	0.221 to 0.346	8.919	< 0.001^*^

## Discussion

The present study yielded three key findings: (1) the TyG index and its modified variants were significantly elevated in patients with poor glycemic control compared to those with good control; (2) the TyG index and its modified variants exhibited significant associations with glycemic control in T2DM; and (3) the TyG index demonstrated superior predictive capability for poor glycemic control compared to the modified indices.

The TyG index, derived from fasting blood glucose and fasting triglyceride levels, is a well-established surrogate marker of IR [12,20]. Obesity, a known contributor to IR, is often assessed using indices such as BMI, WC, WHR, and WHtR. Various studies have incorporated these anthropometric measures into TyG-derived indices (TyG-BMI, TyG-WC, TyG-WHtR) to improve IR assessment [14,21]. Consistent with previous studies, our findings indicate that the TyG index and modified indices (TyG-BMI, TyG-WHR, TyG-WC, and TyG-WHtR) were significantly elevated in T2DM patients with poor glycemic control. These findings align with studies by Hameed [15], Selvi et al. [16], and Timalsina et al. [17], which reported increased TyG index, TyG-BMI, and TyG-WC in T2DM patients with poor glycemic control. However, these studies did not assess TyG-WHR and TyG-WHtR. Furthermore, in our study, both the TyG index and its modified indices exhibited significant positive correlations with HbA1c. This aligns with prior findings by Hameed [15] and Selvi et al. [16], who reported a positive correlation between HbA1c and TyG-derived indices. Multiple prospective studies have demonstrated a correlation between the TyG index and the occurrence of new-onset diabetes mellitus [22,23].

To assess the predictive performance of the TyG index and its variants for poor glycemic control, we conducted an ROC curve analysis. The TyG index exhibited the highest AUC (0.906), followed by TyG-WHR (0.772), TyG-WC (0.631), TyG-WHtR (0.622), and TyG-BMI (0.579). The AUC for the TyG index was significantly higher than that of the other TyG-derived indices. Our findings align with those of a cross-sectional study in Iraq, where the TyG index had the highest AUC (0.839) for predicting poor glycemic control, followed by TyG-WC (0.710) and TyG-BMI (0.651) [15]. Similarly, Selvi et al. [16] and Timalsina et al. [17] reported that the TyG index exhibited superior predictive ability for poor glycemic control compared to other indices. However, contrasting evidence exists. Er et al. [24] found that TyG-BMI and TyG-WC demonstrated greater accuracy in predicting diabetes risk in a Korean cohort, while Xuan et al. [25] suggested that TyG-WHtR outperformed other indices in identifying individuals at risk for T2DM. Ke et al. [21] also reported that among elderly Chinese individuals with normal weight, TyG-WHtR was less strongly associated with T2DM compared to the TyG index alone. These discrepancies highlight potential variations in TyG-derived index performance across different populations.

Our study determined an optimal TyG index cut-off of 5.18 for predicting poor glycemic control in T2DM, with a sensitivity of 79.2% and specificity of 88.8%. Notably, this value differs from prior studies, such as Timalsina et al. [17], who reported a cut-off of ≥9.12 (sensitivity: 86.1%, specificity: 61.5%), and Flake et al. [26], who identified a cut-off of >8.4 (sensitivity: 92.5%, specificity: 47.1%). The reason for the significant variation in the optimal cut-off value of the TyG index for predicting poor glycemic control is that Timalsina et al. [17] and Flake et al. [26] used a slightly different formula to calculate the TyG index: Ln [fasting triglyceride (mg/dL) × fasting glucose (mg/dL)/2].

The precise mechanism underlying the association between the TyG index and T2DM remains unclear. However, several hypotheses have been proposed. One suggests that hepatic gluconeogenesis is stimulated by glycerol and fatty acids, which are products of triglyceride lipolysis [27]. Additionally, elevated triglycerides in pancreatic islets can impair glucose metabolism, leading to β-cell dysfunction and IR [28]. Given that the TyG index integrates both fasting glucose and triglycerides, it may reflect dual aspects of IR: hepatic IR through fasting glucose and adipose tissue IR through triglycerides [29,30]. Since IR is a primary driver of T2DM pathophysiology, this may explain the robust predictive capability of the TyG index in glycemic control assessment.

While HbA1c remains the gold standard for assessing glycemic control, its cost and limited availability in resource-constrained settings present challenges. In contrast, the TyG index, derived from fasting glucose and triglyceride levels, offers a more accessible and cost-effective alternative for glycemic assessment in primary healthcare settings.

Limitations

Several limitations should be acknowledged. First, the study lacks data on dietary intake, physical activity, and diabetes-related complications, which could influence glycemic control. Second, the cross-sectional study design prevents the establishment of causality. Third, key confounding factors such as age and comorbidities were not controlled for, which may have influenced the findings. Lastly, further large-scale studies employing cross-sectional, case-control, or longitudinal designs are necessary to validate the reliability of the TyG index and its modified indices as indicators of glycemic control.

## Conclusions

The TyG index exhibited superior efficacy compared to other modified indices, including TyG-BMI, TyG-WHR, TyG-WC, and TyG-WHtR, in identifying suboptimal glycemic control in individuals with T2DM. Due to its accessibility and cost-effectiveness, the TyG index may serve as a viable alternative for evaluating glycemic control in resource-limited settings where HbA1c testing is not feasible. Future research should aim to validate these findings through longitudinal studies involving larger and more diverse populations to evaluate the long-term predictive value of the TyG index in glycemic control. Furthermore, investigating the integration of the TyG index with other biomarkers or machine-learning models may augment its clinical utility. Additional studies are warranted to assess its applicability in non-diabetic individuals at elevated risk of IR, which could facilitate early disease detection and the development of prevention strategies.
